# Rationale for Testing TP53 Mutations in Thyroid Cancer—Original Data and Meta-Analysis

**DOI:** 10.3390/ijms26031035

**Published:** 2025-01-25

**Authors:** Katarzyna Lacka, Adam Maciejewski, Piotr Tyburski, Ewa Manuszewska-Jopek, Przemysław Majewski, Barbara Więckowska

**Affiliations:** 1 Department of Endocrinology, Metabolism and Internal Medicine, Poznan University of Medical Sciences, 60-355 Poznan, Poland; 2 Student Scientific Society, Poznan University of Medical Sciences, 60-806 Poznan, Poland; 3 Outpatients Unit for Endocrine Diseases, 60-355 Poznan, Poland; 4 Department of Clinical Pathomorphology, Poznan University of Medical Sciences, 60-355 Poznan, Poland; 5 Department of Computer Science and Statistics, Poznan University of Medical Science, 60-806 Poznan, Poland

**Keywords:** thyroid cancer, p53, meta-analysis, mutation, immunohistochemistry

## Abstract

The p53 protein is a tumor-suppressing transcription factor that is critical in tumorigenesis. While *TP53* mutations are rare in differentiated thyroid cancer (DTC), they are significantly more common in anaplastic thyroid cancer (ATC). This study presents original results and a meta-analysis reevaluating the prognostic value of *TP53* mutations in thyroid cancer, including surrogate markers such as immunohistochemical p53 expression and serum p53-Abs levels. *TP53* mutations were analyzed using SSSP and direct sequencing in a DTC group (15 patients), an ATC group (3 patients), and a control group (25 patients). The immunohistochemical p53 expression was assessed in tissue samples. A meta-analysis of 14 eligible studies identified through the PubMed, Scopus, Google Scholar, and Cochrane databases was conducted. Our results showed *TP53* mutations in all ATC cases, 6.67% of DTC cases (1 out of 15), and none in the control group. Immunohistochemical p53 overexpression was observed in 4 out of 15 DTC (26.67%) and all ATC cases but absent in controls. A meta-analysis confirmed that *TP53* mutations are significantly more frequent in ATC than controls (OR 8.95; 95% CI: 1.36–58.70; *p* = 0.02) but not in DTC vs. controls (OR 1.87; 95% CI: 0.53–6.58; *p* = 0.33). p53 overexpression was significantly higher in both DTC and ATC vs. controls (OR 7.99; 95% CI: 5.11–12.51; *p* < 0.01 and OR 64.37; 95% CI: 27.28–151.89; *p* < 0.01, respectively). The serum p53-Abs positivity was also elevated in patients with PTC vs. controls (OR 2.07; 95% CI: 1.24–3.47; *p* < 0.01). *TP53* mutations are frequent events in the pathogenesis of ATC. In DTC, further prospective studies are needed to determine the prognostic value of *TP53* mutations and related surrogate markers (immunohistochemical p53 expression, p53-Abs positivity).

## 1. Introduction

The p53 protein is among the most important molecules in tumorigenesis. Mutations in this tumor-suppressing transcription factor can initiate cancer formation or contribute to further tumor progression. *TP53* mutations are also associated with resistance to therapy, including chemo- and radio-therapy [1]. The role of p53 has been demonstrated in various cancer types [2,3,4,5]. *TP53* mutations are markers of poor prognosis in multiple tumor types, including thyroid cancer [6]. p53 influences numerous aspects of cell growth and metabolism, such as apoptosis, DNA repair, genome stability, and antioxidative mechanisms [7]. This transcription factor is activated in response to cellular stress and can induce diverse responses, e.g., cell cycle arrest, DNA repair, senescence, or apoptosis, depending on multiple factors [4]. It is estimated that *TP53* is one of the most frequently mutated genes in malignancies, with *TP53* mutations being present in over 50% of all tumors [7]. However, these frequencies vary among different cancer types. It may be as high as 89% in small cell lung cancer and 73% in colorectal cancer, but the rates are much lower in some other malignancies, including thyroid cancer (~19%) and cervical cancer (20%) [6]. The occurrence of *TP53* mutations is induced by environmental factors (carcinogens). Correlations exist between specific carcinogens and mutation types [6]. The p53 protein is an important player not only in cancer pathogenesis but also in some aspects of fertility, the immune system and its response to bacterial infections, autoimmunity (including the pathogenesis of autoimmune thyroid diseases), metabolism, and senescence [4,8].

Thyroid cancer is the most common endocrine-related malignancy. Additionally, data show a significant increase in its incidence over the past few decades. Among women, it is now one of the top 10 most frequent malignancies, a trend largely attributed to improvements in diagnostics [9]. The most common type of thyroid cancer is well-differentiated malignant neoplasm arising from thyrocytes, known as differentiated thyroid cancer (DTC), the main subtypes of which are papillary thyroid carcinoma (PTC, responsible for about 90% of all thyroid malignancies), follicular thyroid carcinoma (FTC, 4%), and oncocytic carcinoma of the thyroid (OTC, 2%). Follicular-derived high-grade carcinomas are relatively rare, including poorly differentiated thyroid carcinoma (PDTC) and anaplastic follicular cell-derived thyroid carcinoma (ATC, 1%). Medullary thyroid carcinoma (MTC), the only thyroid cancer derived from C cells, is also infrequent (2%) [9]. *TP53* mutations are generally considered late events that are responsible for progression from *BRAF*-mutated PTC to PDTC and ATC [10]. The frequency of *TP53* mutations is postulated to be low in DTC, estimated at 26% in PDTC and 80% in ATC [11]. However, there are proofs that *TP53* malfunction may promote the progression of PTC via different mechanisms, including action through non-coding lncRNAs and miRNAs [12].

Therefore, the aim of this meta-analysis is to reassess the potential role of *TP53* mutation markers in the course of thyroid cancer, with a special emphasis on differentiated thyroid cancer, which presents a significant epidemiological challenge. Specifically, this analysis seeks to determine the rationale and utility of testing for *TP53* mutations in well-differentiated thyroid cancer. Furthermore, our own results on *TP53* gene mutations and p53 immunohistochemical expression in thyroid cancer will be presented and included in the meta-analysis.

## 2. Results

### 2.1. Original Study

IHC staining for p53 was present in at least 10% of the tumor cell nuclei (positive) in 4 out of 15 of the DTC cases (26.67%, including 3 out of 13 PTC—23.08%—and 1 out of 2 FTC—50%) and in all ATC cases (3 out of 3—100%). In contrast, none of the control group (follicular adenoma and normal thyroid tissue) showed positive results for p53 IHC staining (0 out of 15 and 0 out of 10, respectively). Example figures illustrating immunohistochemistry (IHC) for p53 are provided in Supplementary Figures S1–S3.

Comparable results were obtained in the *TP53* gene mutation analysis. Mutations were confirmed in all ATC cases (three out of three—100%). However, it was a rare event among the patients with DTC (2 out of 15—13.33%), where one patient diagnosed with PTC (1/13—7.69%) and one from the FTC group (1 out of 2—50%) were confirmed positive. *TP53* mutations were absent in the control group (0/15 of follicular adenomas and 0/10 of normal thyroid tissue). Five *TP53* gene mutations were found: one in exon 5, one in exon 6, and three in exon 8. Table 1 shows the clinical data of the patients with and without detected mutations.

### 2.2. Meta-Analysis

Finally, 14 articles and our own results were included in the meta-analysis [13,14,15,16,17,18,19,20,21,22,23,24,25,26]. Seven out of the 15 studies were conducted on the Caucasian population (Italy, Germany, France, Poland, Turkey, USA), and 6 out of the 15 studies were conducted on the Asian population (China, Japan, Taiwan). They were all published between 1993 and 2021.

### 2.3. TP53 Mutations

Six published papers analyzed the incidence of *TP53* mutations among patients with thyroid cancer and the control group. Moreover, our own results have been included. Among the studied patients, there are 278 patients with DTC (including 174 PTC, 65 FTC, and 39 with no defined type), 31 with PDTC, and 22 with ATC. In the control group, there were 139 samples included (normal healthy thyroid, multinodular goiter, or follicular adenoma). Exons 5–8 or exons 5–9 were analyzed as the most frequent sites of mutations. Different DNA sequencing techniques were used (single-strand conformation polymorphism analysis, direct sequencing, or NGS method). The *TP53* mutation frequencies varied from 0 to 25% in the DTC group (6.1% for the whole DTC group), 0 to 17% in the PDTC group (16.1% for the whole PDTC group), and from 0 to 100% in the patients with ATC (22.7% for the whole ATC group) vs. 0% among the controls. There are three studies from European countries (two from Italy and one from Poland), three papers from Asia (China, Taiwan, and Saudi Arabia), and one from the United States.

#### 2.3.1. ATC vs. Control Group

**Figure 1:**
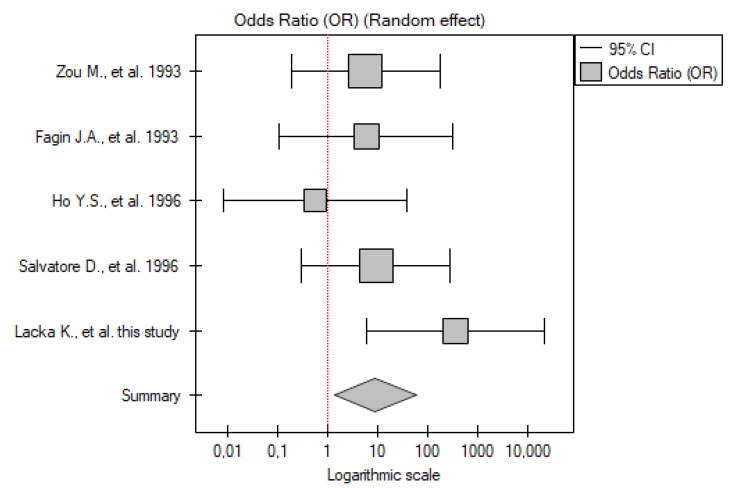
Meta-analysis forest plot of *TP53* gene mutation frequency in ATC vs. control group [15,16,17,23].

Five studies have been included in the analysis (including our own results). The total number of patients included was 22 across all papers (from three to six patients with ATC in each study), with 82 participants in the control group. Meta-analysis showed that the *TP53* mutation frequency was significantly higher among the patients compared to the control group (OR 8.95; 95% CI 1.36–58.70; *p* = 0.02) (Figure 1). A low level of between-studies heterogeneity was found, with there being no statistically significant heterogeneity (I^2^ = 18.83%, *p* = 0.29).

#### 2.3.2. PDTC vs. Control Group

**Figure 2:**
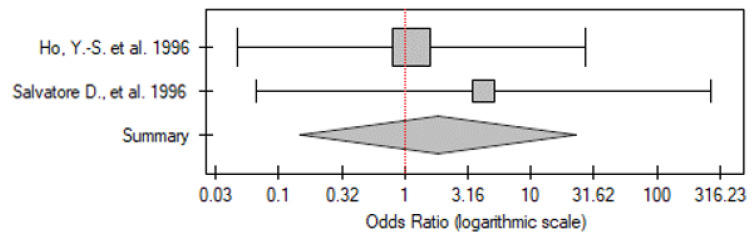
Meta-analysis forest plot of *TP53* gene mutation frequency in PDTC vs. control group [16,23].

Only two of the papers analyzed patients with PDTC vs. the control group (31 vs. 12 participants, respectively). In both studies, there was no significant difference between the patients and controls. After analysing the results of these two papers together, no significant differences were found between patients and controls (OR 1.83; 95% CI 0.15–22.74; *p* = 0.64) (Figure 2). No statistically significant heterogeneity was found (I^2^ = 0%, *p* = 0.62).

#### 2.3.3. DTC vs. Control Group

**Figure 3:**
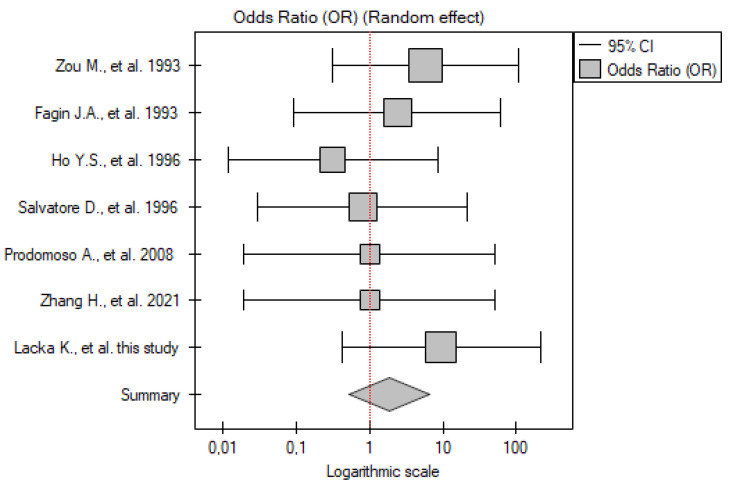
Meta-analysis forest plot of *TP53* gene mutation frequency in DTC vs. control group [13,14,15,16,17,23].

Seven papers were included in the analysis (including our own results). The total number of patients with DTC was 278, and there were 82 participants in the control group. Some between-study variability can be observed, but no study showed significant differences between the patients and controls. The meta-analysis of these results also did not show significant differences between the patients with DTC and the control group (OR 1.87; 95% CI 0.53–6.58; *p* = 0.33) (Figure 3). No statistically significant heterogeneity was found (I^2^ = 0%, *p* = 0.78).

#### 2.3.4. PTC vs. Control Group

**Figure 4:**
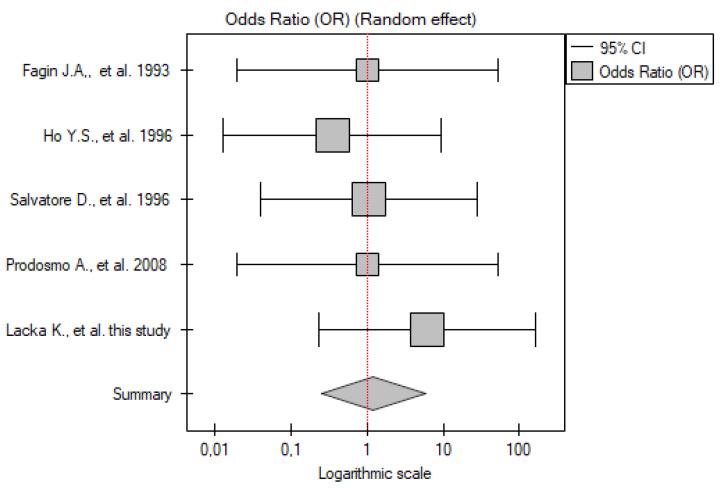
Meta-analysis forest plot of *TP53* gene mutation frequency in PTC vs. control group [14,15,16,23].

In five studies, it was possible to extract data specifically on PTC from the DTC group (or only patients with PTC were included, as in Prodosmo et al. [14]). The meta-analysis included 174 patients and 131 controls. Similar to the DTC group, none of the studies included showed significant differences between the patients and controls. The meta-analysis of these results also did not show significant differences between the patients with PTC and the control group (OR 1.21; 95% CI 0.25–5.82; *p* = 0.81) (Figure 4). No statistically significant heterogeneity was found (I^2^ = 0%, *p* = 0.83).

#### 2.3.5. FTC vs. Control Group

**Figure 5:**
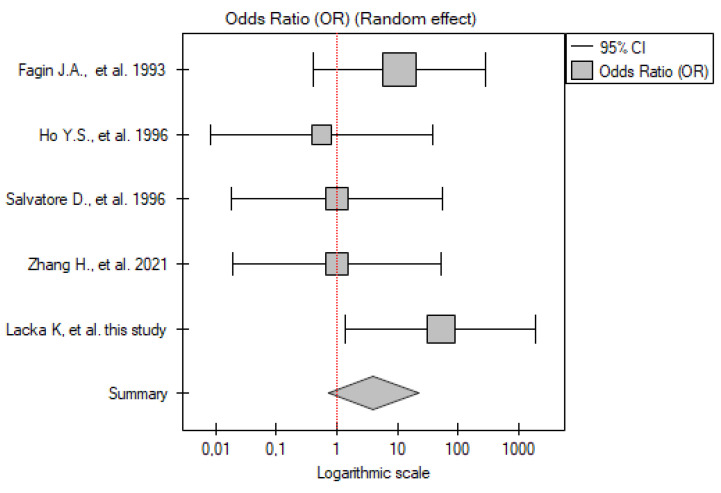
Meta-analysis forest plot of *TP53* gene mutation frequency in FTC vs. control group [13,15,16,23].

In five studies, it was possible to extract data specifically on FTC from the DTC group (or only patients with FTC were included, as in Zhang H et al. [13]). Sixty-five patients with FTC and 70 controls were included in the meta-analysis. Our results showed a significantly higher frequency of *TP53* mutations in the patients with FTC compared to the control group. However, the difference was not significant in other included studies. The meta-analysis of these results did not show significant differences between the patients with FTC and the control group (OR 4.01; 95% CI 0.74–21.81; *p* = 0.11) (Figure 5). No statistically significant heterogeneity was observed between the studies (I^2^ = 1.1%, *p* = 0.40).

When PTC is compared to FTC, the latter has a higher frequency of *TP53* mutations. However, in both groups, there is no significant difference when compared to healthy controls.

### 2.4. IHC

Six published papers analyzed the expression of the p53 protein in the thyroid samples (a surrogate marker of *TP53* mutations) of patients with thyroid cancer vs. the control group. Moreover, our results have been included in the meta-analysis. Among the patients studied, there were 445 patients with DTC (including 275 PTC, 121 FTC, and 49 with no defined type), 54 with PDTC, and 122 with ATC. The control group included 370 tissue samples (normal thyroid, multinodular goitre, follicular adenoma, or Graves disease). The p53-positive results of the IHC staining varied from 0 to 74% in the DTC group (48.3% for the whole DTC group), from 16 to 93% in the PDTC group (57.4% for the whole PDTC group), and from 53 to 100% in the ATC group (64.8% for the whole ATC group) vs. from 0 to 22% among healthy controls (9.8% among all thyroid cancer-free controls). There are three studies from European countries (Germany, Poland and France), three from Asia (China, Japan and Taiwan), and one from South America (Brazil).

#### 2.4.1. ATC vs. Control Group

**Figure 6:**
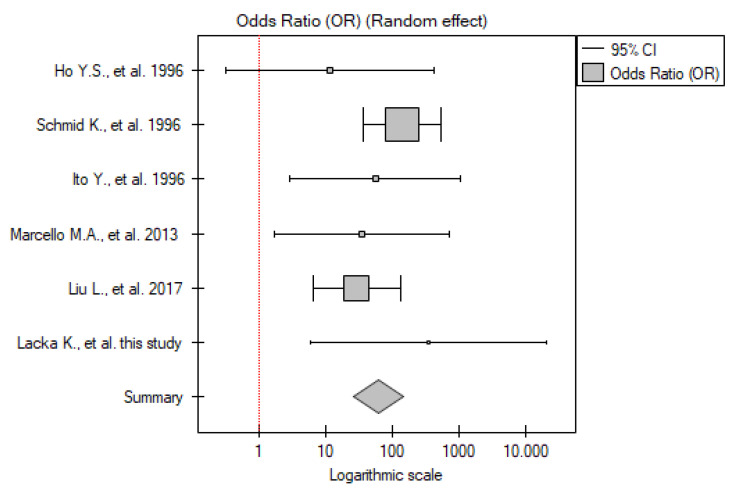
Meta-analysis forest plot of immunohistochemical p53 overexpression frequency in ATC vs. control group [18,19,21,22,23].

Six studies have been included in the analysis (including our results). There were 122 patients and 249 controls analyzed. In all but one paper [23], p53 overexpression was significantly more frequent in the patients than the controls. Meta-analysis also showed that the p53 overexpression frequency was significantly higher among the patients compared to the control group (OR 64.37; 95% CI 27.28–151.89; *p* < 0.01) (Figure 6). No statistically significant heterogeneity was found (I^2^ = 0%, *p* = 0.54).

#### 2.4.2. PDTC vs. Control Group

**Figure 7:**
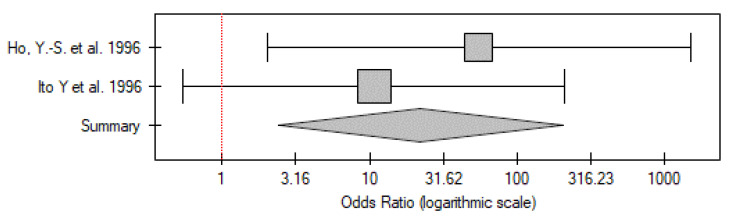
Meta-analysis forest plot of immunohistochemical p53 overexpression frequency in PDTC vs. control group [21,23].

IHC p53 expression was assessed in the PDTC group in only two studies (which included 54 patients and 27 controls). When analyzed together, the results confirmed that the frequency of p53 overexpression was significantly higher in the patients compared to the control group (OR 22.27; 95% CI 2.44–203.23; *p* < 0.01) (Figure 7). No statistically significant heterogeneity was found (I^2^ = 0%, *p* = 0.47).

#### 2.4.3. DTC vs. Control Group

**Figure 8:**
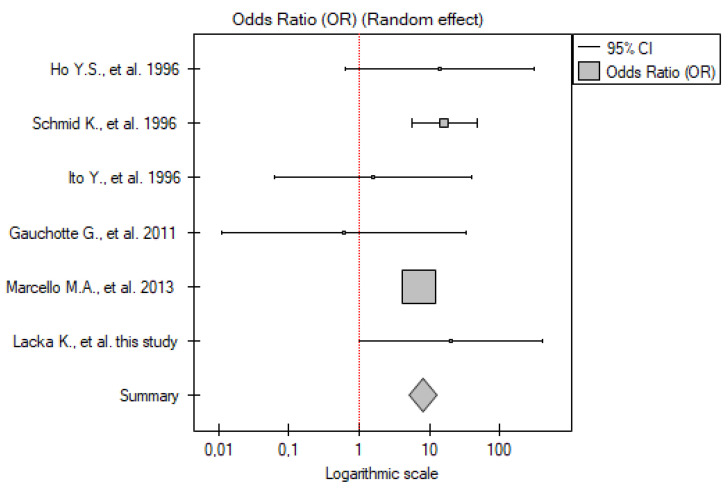
Meta-analysis forest plot of immunohistochemical p53 overexpression frequency in DTC vs. control group [18,20,21,22,23].

Six studies have been included in the analysis (including our results). The total number of patients was 445 vs. 323 in the control group. Meta-analysis showed that the p53 overexpression frequency was significantly higher among the patients with DTC compared to the control group (OR 7.99; 95% CI 5.11–12.51; *p* < 0.01) (Figure 8). No statistically significant heterogeneity was found (I^2^ = 0%, *p* = 0.42).

#### 2.4.4. PTC vs. Control Group

**Figure 9:**
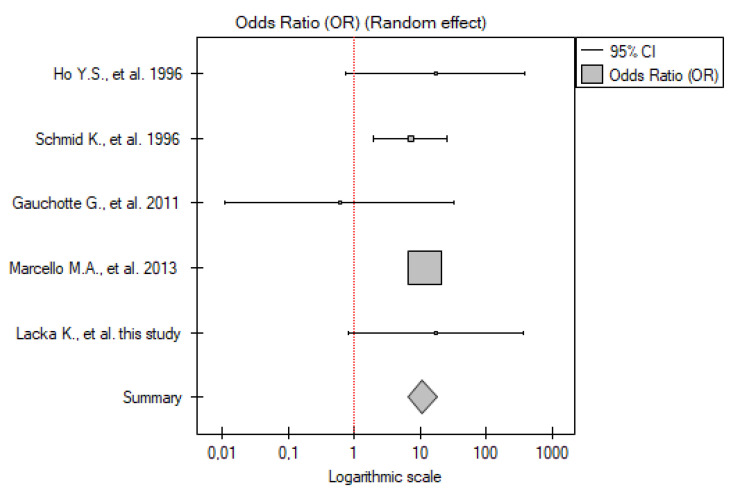
Meta-analysis forest plot of immunohistochemical p53 overexpression frequency in PTC vs. control group [18,20,22,23].

In the case of five of the papers, it was possible to extract data specifically on PTC from that for the DTC group (or only patients with PTC were included, as in Gauchotte G. et al.) [20]. The meta-analysis comprised 275 patients and 298 controls. Similarly to the results for the whole DTC group, the meta-analysis showed that the p53 overexpression frequency was significantly higher among the patients compared to the control group (OR 10.77; 95% CI 6.55–17.73; *p* < 0.01) (Figure 9). No statistically significant heterogeneity was found (I^2^ = 0%, *p* = 0.61).

#### 2.4.5. FTC vs. Control Group

**Figure 10:**
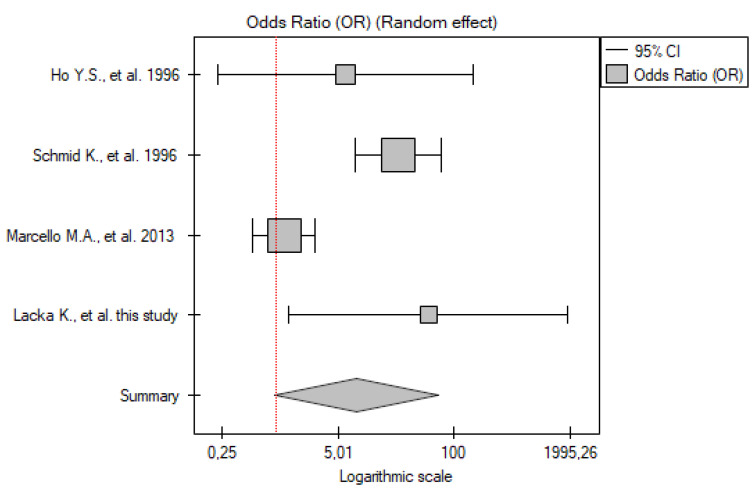
Meta-analysis forest plot of immunohistochemical p53 overexpression frequency in FTC vs. control group [18,22,23].

In the case of four of the studies, it was possible to extract data specifically on FTC from the DTC group. There were 121 patients with FTC and 288 controls. A meta-analysis showed that p53 overexpression was more frequent among the patients compared to the control group, although only with borderline significance (OR 7.99; 95% CI 0.97–65.92; *p* = 0.05) (Figure 10). Despite the relatively high level of heterogeneity (I^2^ = 85.04%, *p* < 0.01), in all papers, the frequency of p53 overexpression was higher among the patients than in the control group.

### 2.5. P53 Ab

#### PTC vs. Control Group

**Figure 11:**
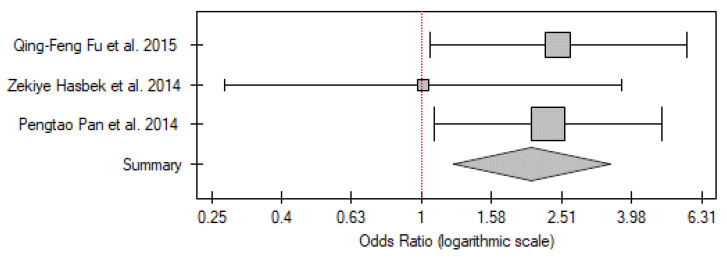
Meta-analysis forest plot of P53 antibodies positivity in PTC vs. control group [24,25,26].

The anti-p53 antibody levels of patients with thyroid cancer vs. the control group were assessed by only three studies. Of all available publications all analyzed PTC, there were no papers on ATC or FTC types. The number of participants included in the meta-analysis was 562 patients with PTC and 230 healthy controls. Two of the three papers found p53 antibodies to be significantly more frequent in the patients, and the overall OR was 2.07 (95% CI 1.24–3.47; *p* < 0.01) (Figure 11). No statistically significant heterogeneity was found (I^2^ = 0%, *p* = 0.50).

## 3. Discussion

The *TP53* gene, located at 17p13.1, comprises 11 exons and 393 amino acid residues. Mutations in the *TP53* gene can be found across all its exons, but most are located in the DNA-binding domain (exons 5–8), which affects the ability of the p53 transcription factor to bind to target genes [7]. Among the various mutation types, missense mutations are the most common, making up about 80% of mutations [27]. Mutations of *TP53* can lead not only to a loss of p53’s protective function but also to a gain of function that promotes tumour progression by, among other methods, altering the transcription of genes other than those regulated by the wild-type gene [7].

Although the general importance of the *TP53* gene mutations in cancer pathogenesis is well known, its frequency and prognostic role in specific tumors is still a matter of debate. In prostate cancer, a large number of studies have confirmed the association between *TP53* mutations and faster disease progression [28]. Similarly, it has been proven that *TP53* mutations determine a worse prognosis and shorter overall survival in acute myeloid leukemia [29]. *TP53* gene mutations are also thought to be an important prognostic factor for survival in head and neck squamous cell carcinoma [30]. On the contrary, in a meta-analysis of patients with metastatic colorectal cancer, its prognostic value was not confirmed [31].

Thyroid cancer development involves multiple molecular events. *BRAF* mutations and *RET*/*PTC* rearrangements are the most frequent molecular events in PTC. Some researchers suggest that *TP53* mutation might be the sole molecular event that transforms normal thyroid cells into ATC [11,32]. However, the prevailing models for the development of ATC and PDTC suggest that they evolve from DTC due to cell selection and the gain of further mutation [32]. As shown by Landa et al., *TP53* mutations are much more often observed in ATCs compared to PDTCs and, therefore, may facilitate the distinction between these two categories [32].

In thyroid cancers, especially ATC, *TP53* mutations most frequently occur in exons 5–9, with codon 273 being the most common hotspot [11]. In general, hotspot areas are similar to those found in other cancers, including codons R175, R245, R248, R249, R273, and R282 [11]. The wild-type p53 in thyrocytes seems to directly activate the promoter of the sodium-iodide symporter (NIS) gene, maintaining its proper expression. Therefore, *TP53* mutations can affect sensitivity to radioiodine treatment [19].

Our own results and meta-analysis confirmed that *TP53* mutation is common in ATC. Even if some studies fail to confirm the significantly higher frequency of *TP53* mutations in this undifferentiated thyroid cancer group, it may largely be due to the small sample size. The difference is much less evident in the case of PDTC, although, again, the small sample size makes it challenging to come to conclusions. In the case of DTC, as expected, the frequency of *TP53* mutations is low and not significantly higher than in the control group. However, still, in this type of thyroid cancer, which has the best prognosis, there are cases with *TP53* mutations, and their frequency may be as high as 25% in some cohorts [11]. To explain this, a closer look at these patients and their clinical course would be required. Probably, these DTCs with *TP53* mutations are cases with an unfavorable response to the treatment. It cannot be excluded that gene mutations are detectable in foci of dedifferentiation [33].

The level and activity of the p53 protein depend not only on its expression but also on various post-translational modifications like ubiquitination, phosphorylation, acetylation, and methylation, as well as degradation. *TP53* mutations can cause an increase in p53 protein accumulation, although the exact mechanism of this is still under study. These two parameters—*TP53* mutations and p53 protein levels—are not always directly correlated [18,34]. In some malignancies, such as B-cell lymphoma, IHC staining is not an accurate surrogate marker for gene mutations [35]. On the contrary, it was proven useful at least for screening in some solid tumors [36]. In endometrial cancer [37], colorectal cancer [38], and various solid tumors [39], a high correlation between *TP53* mutations and p53 immunohistochemistry (IHC) has been established (concordance rate > 90%), making p53 IHC a reliable surrogate marker for *TP53* status. Unfortunately, no precise data exist for thyroid cancer. It must be taken into account that the actual concordance rate could be significantly lower in earlier studies when less accurate methods were used to assess *TP53* mutations and p53 expression [40].

Many studies have highlighted the role of p53 as a prognostic marker in thyroid cancer. It is often linked to worse outcomes or more advanced disease features (such as a larger tumor size and the presence of lymph node metastasis), although there are some contradictory findings [18,41,42]. In a 2021 study, Martins and colleagues discovered that the co-expression of p53 and MDM2 tends to be a marker of less aggressive clinical outcomes, contrasting with earlier observations [43,44]. Further studies are needed to fully explain the prognostic value of the IHC expression of p53 in tumor tissues. Observations from other malignancies may also shed some light on this topic.

We and others (as shown in the meta-analysis) showed that p53 overexpression is frequent not only in ATC samples but also in well-differentiated thyroid cancer tissues (with a mean frequency of almost 50%). These frequencies were significantly higher compared to the control group. Even though *TP53* mutations are primarily seen in PDTC and ATC, p53 overexpression can also be observed in DTC, similar to what occurs in the early stages of other tumors. Some heterogeneity of the results can be attributed to the patients included, but there are also methodological discrepancies and a lack of established criteria for overexpression (different thresholds used in various publications). p53 overexpression is substantially more frequent than *TP53* mutations (especially in DTC). This confirms that not only gene mutation but also the deregulation of other processes may be the reason for this. It should be also highlighted that it is now established that not only p53 overexpression but also other patterns of p53 IHC expression, such as the null pattern and, more rarely, cytoplasmic positive staining, can be associated with gene mutations [38,39]. Some sources confirm that, in the ATC group, both p53 overexpression (positive abnormal pattern) and, even more frequently, a loss of expression are observed [45].

The search for new cancer markers is ongoing, and one critical feature of a good marker is that it is simple and easy to measure. In this respect, testing for p53 antibodies (p53-Abs) seems attractive. The mutated form of p53 is more stable than the wild-type, leading to the accumulation of p53 and an increase in p53-Abs formation. Notably, both anti-mutated p53 and anti-wild-type p53-Abs can be detected [46]. There is no absolute correlation between *TP53* mutations and p53-Abs, but they are postulated as surrogate marker of gene mutation [47,48]. Depending on the detection method and study population, p53-Abs can be found in 11–19% of patients with PTC vs. 8–11% in healthy individuals [24,25,26].

Overall, our analysis has confirmed that, even in patients with well-differentiated thyroid cancer, p53-Abs is significantly more frequent compared to patients without thyroid malignancy, and this relatively simple laboratory marker has some potential to improve diagnostics in this group. It is also expected that p53-Abs positivity is associated with a poor clinicopathological course in patients with DTC (e.x. multifocality, lymph node metastasis, higher TNM stage) and a worse outcome [24]. This parameter has been used more extensively as a biomarker in other, more aggressive tumors, including colorectal cancer, other gastrointestinal cancers, and lung cancer [49,50]. A broader meta-analysis confirms that the presence of p53-Abs across different solid tumors is associated with a worse prognosis, making it a potential negative prognostic marker [46]. The p53 antibodies seem more reliable when used alongside other antibodies in a panel.

Regardless of the detection method, cases with mutated *TP53* are associated with more aggressive behavior, including a higher risk of metastasis, angioinvasion, dedifferentiation, and a general poor response to treatment [51,52,53]. However, defective p53 can be a therapeutic target. Although targeting mutated *TP53* in therapy is challenging, there are emerging possibilities, such as p53-targeting cancer vaccines, MDM2 inhibitors (a negative regulator of p53), and monoclonal antibodies that target mutated p53 epitopes [6,54,55].

There are several limitations to our study. The central issue appears to be the relatively small sample size of the included studies. This may be justified in the case of ATC due to its low incidence, but it raises concerns when DTC is analyzed. Secondly, many papers included in the meta-analysis are not up-to-date, with more than half being published over 20 years ago, and there is a lack of newer studies. Since then, diagnostic methods have improved, not only in genetic studies but also in immunohistochemical staining. A more nuanced approach should be adopted, beyond the simple positive/negative p53 or scored p53 overexpression used in earlier publications (including most of those presented in the meta-analysis). More advanced scoring systems, such as the H-score and four-tier expression pattern assessment, should be implemented and correlated with up-to-date sequencing results.

## 4. Material and Methods

### 4.1. Original Study

#### 4.1.1. Patients

The study group consisted of 15 patients diagnosed with DTC, including 13 cases of PTC and 2 cases of FTC, as well as 3 patients with ATC. The control group included 15 patients with follicular adenoma and 10 patients with normal thyroid without nodules or other known thyroid pathologies (who had partial thyroid removal during neck surgery for other reasons). All patients in the study group underwent thyroidectomy due to preoperatively diagnosed thyroid lesions (thyroid ultrasonography and fine needle aspiration biopsy). Among the patients, there were 25 women and 8 men. In the control group, thyroidectomy or partial thyroid removal was performed. Thyroid tissue samples were collected from Polish patients operated on in surgical departments and further analyzed at the Department of Pathomorphology of the Poznan University of Medical Sciences.

#### 4.1.2. Mutation Detection

DNA was isolated from frozen thyroid specimens using the phenol method with proteinase K. The amount and quality of isolated DNA were further assessed, confirming good yield. Polymerase chain reaction (PCR) was used to amplify exons 5–8. PCR conditions for genotyping exons 5–8 were as follows: (1) predenaturation at 94 °C for 3 min—exons 6 and 7, 5 min—exons 5 and 8; (2) amplification 35× (denaturation at 94 °C for 30 s—exons 6 and 7, 60 s—exons 5 and 8; annealing at 64 °C for 90 s—exons 7, at 72 °C for 60 s—exons 5 and 8, at 72 °C for 90 s—exon 6; elongation at 72 °C for 120 s—exons 5 and 8, 180 s—exons 6 and 7); (3) final extension at 72 °C for 5 min for all exons. Starter sequences used:

- Exon 5

P5F 5′CTCTTCCTACAGTACTCCCCTGC 3′

P5R 3′ATCGCTACCACTCGTCGACCCCG 5′;

- Exon 6

P6F 5′ GATTGCTCTTAGGTCTGGCCCCTC 3′

P6R 3′ CCAATTCCCACCAACAGTCACCGG 5′;

- Exon 7

P7F 5′ CTCATCTTGGGCCTGTGTATCTCCTAGG 3′

P7R 3′ CTGAGGTCCAGTCCTCGGTGAACGGT 5′;

- Exon 8

P8F 5′ ACCTGATTTCCTTACTGCCTCTTGC 3′

P8R 3′ TGATTCGCTCCATTCGTTCGTCCTG 5′.

The amplification product was initially analyzed by the single-strand conformation polymorphism (SSCP) technique using polyacrylamide gel electrophoresis (10% polyacrylamide gel, 16 h, 70 V). In cases where SSCP found positive results, direct sequencing was performed to confirm the results (AbiPrism, Applied Biosystems, Foster City, CA, USA).

#### 4.1.3. Immunohistochemistry

The examined tissues, fixed in 4% buffered formalin and embedded in paraffin, were sectioned into 4-micrometer slices and mounted on adhesive-coated slides (SuperFrost^®^ Plus, Menzel Gläser, Epredia, Kalamazoo, MI, USA). Deparaffinization, rehydration, and epitope retrieval were conducted in a water bath (PT LINK, Dako, Agilent Technologies, Santa Clara, CA, USA) at 97 °C (20 min) using high pH EnVision Flex Target Retrieval Solution (Dako). Subsequently, the sections, following a 10 min incubation in wash buffer (Dako), were stained using an automated staining system—Autostainer Link 48 (Dako)—employing the EnVision Flex Mini Kit, with high pH (Link), (Dako, cat. No K8023, Agilent Technologies, Santa Clara, CA, USA) for immunohistochemical staining visualization. For antibody expression analysis, a monoclonal mouse anti-p53 antibody (clone DO-7) in ready-to-use form from Dako was used. The reaction was amplified using a mouse linker (EnVision FLEX + Mouse LINKER). Incubation with the antibody lasted 20 min. The color reaction visualization was carried out using 3,3′-diaminobenzidine tetrachloride. The sections were counterstained with Mayer’s hematoxylin, dehydrated, and mounted with a coverslip using DPX. To confirm the accuracy of staining, each staining procedure included a positive control (tonsil) and a negative control (omitting the primary antibody). An experienced pathologist subsequently evaluated the stained slides. The p53 result was considered positive (+) when at least 10% of the tumor cell nuclei showed a clear positive reaction; the reaction was scored as strongly positive (++/+++) when at least 50% of the tumor cell nuclei showed clear staining. If less than 10% of tumor cell nuclei showed the reaction, p53 expression was considered negative (−).

### 4.2. Meta-Analysis

**Figure 12:**
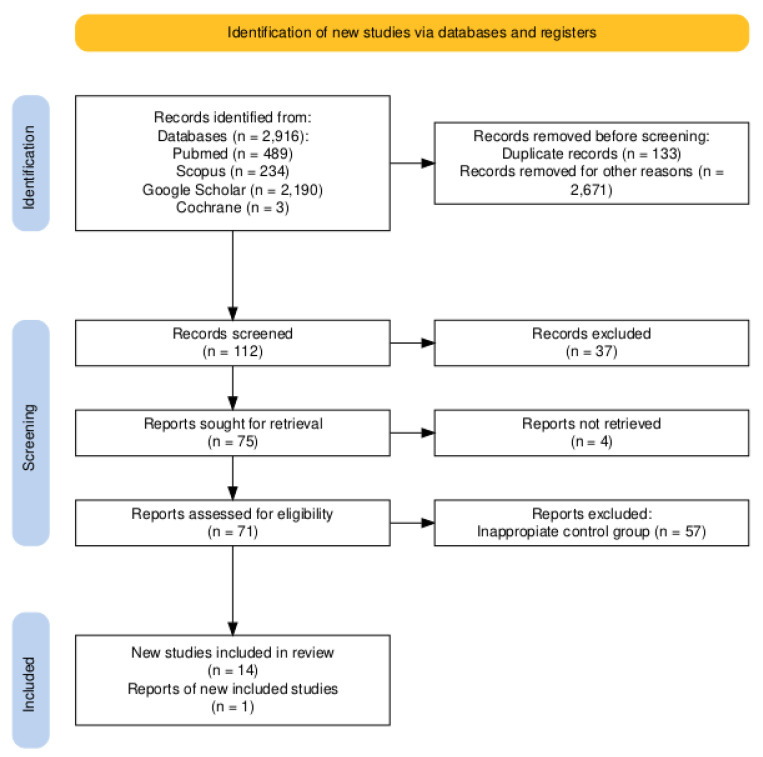
Flowchart of screening of the literature.

The research methodology relied on specific search terms, adhering to the PRISMA 2020 checklist and employing the PRISMA 2020 flow diagram as illustrated in Figure 12. The PRISMA checklist was verified during the review draft. Utilizing PubMed, Scopus, Google Scholar, and Cochrane as primary search engines, the research focused on the terms listed below (‘thyroid carcinoma’, ‘thyroid cancer’, ‘p53 mutation’ and ‘*TP53* mutation’). Two authors independently searched the databases. If any doubts or disagreements arose, the authors discussed the issue to arrive at a final decision. Both randomized controlled trials and non-randomized controlled trials were included, with a requirement that papers be published in English. Following the selection of eligible studies, relevant data were extracted. In designing the inclusion criteria, we utilized the PICO framework: the selected studies had to contain both the research group (patients diagnosed with differentiated/poorly differentiated or undifferentiated thyroid cancer) and the control group (healthy patients/goiters/thyroid adenomas) (population: P). We measured the *TP53* mutation incidence using various methods (*TP53* gene sequencing, IHC staining, serum p53 antibodies level) and the clinical outcome (intervention: I). These parameters were then compared via statistical software between research and control groups (comparison: C). The primary outcomes (O) were measured by comparing the *TP53* mutation incidence and disease development at the study’s conclusion between the research group and the control group. Reviews and case reports or case series, as well as animal or experimental studies, were excluded from the analysis as well as studies without English as their primary language. We also eliminated studies focusing solely on a specific population (e.g., limited to Hobnail variant thyroid carcinoma). The control group had to consist of either healthy volunteers or patients with benign thyroid lesions or other, non-neoplastic thyroid disorders. Two researchers independently conducted the selection process, with subsequent collaboration to reconcile any discrepancies. Furthermore, the authors analyzed the data (tumor type, methodology, p53 mutation incidence) utilizing statistical software. During the identification stage, 489 records were sourced from PubMed, 234 from Scopus, 2190 from Google Scholar, and 3 from Cochrane. Inserted query involved the following terms: ‘thyroid carcinoma’, ‘thyroid cancer’, ‘p53 mutation’, and ‘*TP53* mutation’. After eliminating duplicates and various other data that met the exclusion criteria, 112 records were subjected to screening. Among these, 37 were excluded, and 4 were not retrieved. The remaining 71 full papers underwent further scrutiny—14 articles were included in the analysis: 6 utilizing immunohistochemistry, 6 entailing *TP53* gene mutation assessment, and 3 based on p53 antibody analysis [13,14,15,16,17,18,19,20,21,22,23,24,25,26]. Our own results consisting of one *TP53* gene sequencing study and one immunohistochemistry method study were also added. As a result, the whole meta-analysis consisted of 15 studies.

## 5. Conclusions

*TP53* mutations exhibit potential as both a diagnostic tool and a prognostic marker in thyroid cancer, including patients with DTC. *TP53* mutations are primarily detected in high-grade thyroid cancer. However, when *TP53* mutations or their surrogate markers—IHC expression aberrations or p53-Abs positivity—are identified in patients with DTC, they may serve as negative prognostic indicators, being associated with poor treatment response. Further prospective studies are warranted to elucidate their prognostic significance in real-world settings. Moreover, a comprehensive analysis of the relationship between *TP53* mutations and their surrogate markers, specifically within the context of thyroid cancer, is essential.

## Figures and Tables

**Table 1:** Patient characteristics.

No.	Gender	Age	TNM	p53 IHC Staining	*TP53* Mutation
PTC					
1	F	32	T2N0M0	+	−
2	F	17	T2N0M0	−	−
3	F	17	T1N0M0	−	−
4	F	60	T2N1aM0	−	−
5	F	15	T2N0M0	−	−
6	F	68	T4N1bM0	+	−
7	F	50	T1N0M0	−	−
8	F	75	T2N0M0	−	−
9	F	40	T1N0M0	−	−
10	F	16	T2N0M0	−	−
11	M	40	T4N1bM0	+	+ exon 5
12	F	33	T2N0M0	−	−
13	M	15	T2N1aM1	−	−
FTC					
1	F	18	T1N0M0	−	−
2	F	64	T4N1bM1	+	+ exon 6
ATC					
1	F	73	T4N1bM1	+	+ exon 8
2	M	72	T4N1bM1	+	+ exon 8
3	M	64	T4N1bM1	+	+ exon 8

PTC—papillary thyroid cancer; FTC—follicular thyroid cancer; ATC—anaplastic thyroid cancer.

## Data Availability

The raw data are available from the corresponding author upon request.

## Supplementary Materials

The following supporting information can be downloaded at: https://www.mdpi.com/article/10.3390/ijms26031035/s1.

## Abbreviations

ATC	Anaplastic thyroid carcinoma
CI	Confidence Interval
DTC	Differentiated thyroid cancer
FTC	Follicular thyroid carcinoma
IHC	Immunohistochemistry
MTC	Medullary thyroid carcinoma
NGS	Next-Generation Sequencing
OR	Odds Ratio
OTC	Oncocytic carcinoma of the thyroid
PDTC	Poorly differentiated thyroid carcinoma
PRISMA	Preferred Reporting Items for Systematic Reviews and Meta-Analyses
PTC	Papillary thyroid carcinoma
p53-Abs	p53 antibodies
SSCP	Single-Strand Conformation Polymorphism
TNM	Tumor, Nodes, Metastasis (staging system)
TP53	Tumor protein p53 (gene)

## References

[1] Lim D.V., Woo W.H., Lim J.X., Loh X.Y., Soh H.T., Lim S.Y.A., Lee Z.Y., Yow H.Y., Hamzah S.B., Sellappans R. (2024). Targeting Mutant-p53 for Cancer Treatment: Are We There Yet?. Curr. Mol. Pharmacol..

[2] Donehower L.A., Soussi T., Korkut A., Liu Y., Schultz A., Cardenas M., Li X., Babur O., Hsu T.K., Lichtarge O. (2019). Integrated Analysis of TP53 Gene and Pathway Alterations in The Cancer Genome Atlas. Cell Rep..

[3] Muller P.A., Vousden K.H. (2013). p53 Mutations in Cancer. Nat. Cell Biol..

[4] Levine A.J. (2019). The Many Faces of p53: Something for Everyone. J. Mol. Cell Biol..

[5] Bai B., An X., Qu Q., Liu X., Liu Y., Wei L. (2024). The Clinical Features and Prognostic Implications of the Co-Mutated TP53 Gene in Advanced Non-Small Cell Lung Cancer. Clin. Transl. Oncol..

[6] Chen X., Zhang T., Su W., Dou Z., Zhao D., Jin X., Lei H., Wang J., Xie X., Cheng B. (2022). Mutant p53 in Cancer: From Molecular Mechanism to Therapeutic Modulation. Cell Death Dis..

[7] Zhang C., Liu J., Xu D., Zhang T., Hu W., Feng Z. (2020). Gain-of-Function Mutant p53 in Cancer Progression and Therapy. J. Mol. Cell Biol..

[8] Lacka K., Maciejewski A. (2012). Rola procesu apoptozy w etiopatogenezie autoimmunologicznego zapalenia tarczycy [The Role of Apoptosis in the Etiopathogenesis of Autoimmune Thyroiditis]. Pol. Merkur Lekarski..

[9] Kitahara C.M., Schneider A.B. (2022). Epidemiology of Thyroid Cancer. Cancer Epidemiol. Biomark. Prev..

[10] Quiros R.M., Ding H.G., Gattuso P., Prinz R.A., Xu X. (2005). Evidence That One Subset of Anaplastic Thyroid Carcinomas Are Derived from Papillary Carcinomas Due to BRAF and p53 Mutations. Cancer.

[11] Romei C., Elisei R. (2021). A Narrative Review of Genetic Alterations in Primary Thyroid Epithelial Cancer. Int. J. Mol. Sci..

[12] Wang G., Wei L., Yang H. (2024). p53-Associated miRNAs Repress lncRNA ZFAS1 to Retard the Proliferation of Papillary Thyroid Carcinoma. Endokrynol. Pol..

[13] Zhang H., Zhang Z., Liu X., Duan H., Xiang T., He Q., Su Z., Wu H., Liang Z. (2021). DNA Methylation Haplotype Block Markers Efficiently Discriminate Follicular Thyroid Carcinoma from Follicular Adenoma. J. Clin. Endocrinol. Metab..

[14] Prodosmo A., Giglio S., Moretti S., Mancini F., Barbi F., Avenia N., Di Conza G., Schünemann H.J., Pistola L., Ludovini V. (2008). Analysis of Human MDM4 Variants in Papillary Thyroid Carcinomas Reveals New Potential Markers of Cancer Properties. J. Mol. Med..

[15] Fagin J.A., Matsuo K., Karmakar A., Chen D.L., Tang S.H., Koeffler H.P. (1993). High Prevalence of Mutations of the p53 Gene in Poorly Differentiated Human Thyroid Carcinomas. J. Clin. Investig..

[16] Salvatore D., Celetti A., Fabien N., Paulin C., Martelli M.L., Battaglia C., Califano D., Monaco C., Viglietto G., Santoro M. (1996). Low Frequency of p53 Mutations in Human Thyroid Tumors; p53 and Ras Mutation in Two Out of Fifty-Six Thyroid Tumors. Eur. J. Endocrinol..

[17] Zou M., Shi Y., Farid N.R. (1993). p53 Mutations in All Stages of Thyroid Carcinomas. J. Clin. Endocrinol. Metab..

[18] Marcello M.A., Morari E.C., Cunha L.L., De Nadai Silva A.C., Carraro D.M., Carvalho A.L., Soares F.A., Vassallo J., Ward L.S. (2013). P53 and Expression of Immunological Markers May Identify Early Stage Thyroid Tumors. Clin. Dev. Immunol..

[19] Liu L., Li D., Chen Z., Yang J., Ma Y., Cai H., Shan C., Lv Z., Zhang X. (2017). Wild-Type P53 Induces Sodium/Iodide Symporter Expression Allowing Radioiodide Therapy in Anaplastic Thyroid Cancer. Cell Physiol. Biochem..

[20] Gauchotte G., Philippe C., Lacomme S., Léotard B., Wissler M.P., Allou L., Toussaint B., Klein M., Vignaud J.M., Bressenot A. (2011). BRAF, p53, and SOX2 in Anaplastic Thyroid Carcinoma: Evidence for Multistep Carcinogenesis. Pathology.

[21] Ito Y., Kobayashi T., Takeda T., Komoike Y., Wakasugi E., Tamaki Y., Tsujimoto M., Matsuura N., Monden M. (1996). Expression of p21 (WAF1/CIP1) Protein in Clinical Thyroid Tissues. Br. J. Cancer..

[22] Schmid K.W., Bankfalvi A., Mucke S., Ofner D., Riehemann K., Schroder S., Stucker A., Totsch M., Dockhorn-Dworniczak B. (1996). Possible Relation of p53 and MDM2 Oncoprotein Expression in Thyroid Carcinoma: A Molecular-Pathological and Immunohistochemical Study on Paraffin-Embedded Tissue. Endocr. Pathol..

[23] Ho Y.S., Tseng S.C., Chin T.Y., Hsieh L.L., Lin J.D. (1996). p53 Gene Mutation in Thyroid Carcinoma. Cancer Lett..

[24] Fu Q.F., Pan P.T., Zhou L., Liu X.L., Guo F., Wang L., Sun H. (2015). Clinical Significance of Preoperative Detection of Serum p53 Antibodies and BRAF(V600E) Mutation in Patients with Papillary Thyroid Carcinoma. Int. J. Clin. Exp. Med..

[25] Hasbek Z., Turgut B., Erselcan T. (2014). p53 Antibody: Is It an Indicator of Dedifferentiated Thyroid Cancer?. Ann. Nucl. Med..

[26] Pan P., Han X., Li F., Fu Q., Gao X., Sun H., Wang L. (2014). Detection of Serum p53 Antibodies from Chinese Patients with Papillary Thyroid Carcinoma Using Phage-SP-ELISA: Correlation with Clinical Parameters. Endocrine.

[27] Chiang Y.T., Chien Y.C., Lin Y.H., Wu H.H., Lee D.F., Yu Y.L. (2021). The Function of the Mutant p53-R175H in Cancer. Cancers.

[28] Maddah M.M., Hedayatizadeh-Omran A., Moosazadeh M., Alizadeh-Navaei R. (2024). Evaluation of the Prognostic Role of TP53 Gene Mutations in Prostate Cancer Outcome: A Systematic Review and Meta-Analysis. Clin. Genitourin. Cancer.

[29] Qin G., Han X. (2022). The Prognostic Value of TP53 Mutations in Adult Acute Myeloid Leukemia: A Meta-Analysis. Transfus. Med. Hemother..

[30] Basyuni S., Nugent G., Ferro A., Barker E., Reddin I., Jones O., Lechner M., O’Leary B., Jones T., Masterson L. (2022). Value of p53 Sequencing in the Prognostication of Head and Neck Cancer: A Systematic Review and Meta-Analysis. Sci. Rep..

[31] Ottaiano A., Santorsola M., Capuozzo M., Perri F., Circelli L., Cascella M., Ianniello M., Sabbatino F., Granata V., Izzo F. (2023). The Prognostic Role of p53 Mutations in Metastatic Colorectal Cancer: A Systematic Review and Meta-Analysis. Crit. Rev. Oncol. Hematol..

[32] Landa I., Ibrahimpasic T., Boucai L., Sinha R., Knauf J.A., Shah R.H., Dogan S., Ricarte-Filho J.C., Krishnamoorthy G.P., Xu B. (2016). Genomic and Transcriptomic Hallmarks of Poorly Differentiated and Anaplastic Thyroid Cancers. J. Clin. Investig..

[33] Luo H., Xia X., Kim G.D., Liu Y., Xue Z., Zhang L., Shu Y., Yang T., Chen Y., Zhang S. (2021). Characterizing Dedifferentiation of Thyroid Cancer by Integrated Analysis. Sci. Adv..

[34] Koga T., Hashimoto S., Sugio K., Yoshino I., Nakagawa K., Yonemitsu Y., Sugimachi K., Sueishi K. (2001). Heterogeneous Distribution of P53 Immunoreactivity in Human Lung Adenocarcinoma Correlates with MDM2 Protein Expression, Rather than with P53 Gene Mutation. Int. J. Cancer.

[35] de Haan L.M., de Groen R.A.L., de Groot F.A., Noordenbos T., van Wezel T., van Eijk R., Ruano D., Diepstra A., Koens L., Nicolae-Cristea A. (2024). Real-world routine diagnostic molecular analysis for TP53 mutational status is recommended over p53 immunohistochemistry in B-cell lymphomas. Virchows Arch..

[36] Armbruster H., Schotte T., Götting I., Overkamp M., Granai M., Volmer L.L., Bahlinger V., Matovina S., Koch A., Dannehl D. (2024). Aberrant p53 Immunostaining Patterns in Breast Carcinoma of No Special Type Strongly Correlate with Presence and Type of TP53 Mutations. Virchows Arch..

[37] Vermij L., Léon-Castillo A., Singh N., Powell M.E., Edmondson R.J., Genestie C., Khaw P., Pyman J., McLachlin C.M., Ghatage P. (2022). p53 immunohistochemistry in endometrial cancer: Clinical and molecular correlates in the PORTEC-3 trial. Mod. Pathol..

[38] Osakabe M., Yamada N., Sugimoto R., Uesugi N., Nakao E., Honda M., Yanagawa N., Sugai T. (2024). The Pattern-Based Interpretation of p53 Immunohistochemical Expression as a Surrogate Marker for TP53 Mutations in Colorectal Cancer. Virchows Arch..

[39] Sung Y.N., Kim D., Kim J. (2022). p53 Immunostaining Pattern Is a Useful Surrogate Marker for TP53 Gene Mutations. Diagn. Pathol..

[40] Köbel M., Piskorz A.M., Lee S., Lui S., LePage C., Marass F., Rosenfeld N., Mes Masson A.M., Brenton J.D. (2016). Optimized p53 Immunohistochemistry as an Accurate Predictor of TP53 Mutation in Ovarian Carcinoma. J. Pathol. Clin. Res..

[41] Morita N., Ikeda Y., Takami H. (2008). Clinical Significance of P53 Protein Expression in Papillary Thyroid Carcinoma. World J. Surg..

[42] Abdelhafez D.N., Ayoub M.M., Mahmoud S.A., El Hanbuli H.M. (2023). YAP1 and P53 Expression in Papillary Thyroid Carcinoma. Iran. J. Pathol..

[43] Martins M.B., de Assis Batista F., Marcello M.A., Bufalo N.E., Peres K.C., Morari E.C., Soares F.A., Vassallo J., Ward L.S. (2021). Clinical Utility of the Immunohistochemical Co-Expression of p53 and MDM2 in Thyroid Follicular Lesions. Ann. Diagn Pathol..

[44] Horie S., Maeta H., Endo K., Ueta T., Takashima K., Terada T. (2001). Overexpression of p53 Protein and MDM2 in Papillary Carcinomas of the Thyroid: Correlations with Clinicopathologic Features. Pathol. Int..

[45] Mneimneh W.S., Asa S.L. (2024). Divergent Lineage Markers in Anaplastic Thyroid Carcinoma. Am. J. Surg. Pathol..

[46] Sobhani N., Roviello G., D’Angelo A., Roudi R., Neeli P.K., Generali D. (2021). p53 Antibodies as a Diagnostic Marker for Cancer: A Meta-Analysis. Molecules.

[47] Soussi T. (2000). p53 Antibodies in the Sera of Patients with Various Types of Cancer: A Review. Cancer Res..

[48] Mudry P., Slaby O., Neradil J., Soukalova J., Melichar B., Sachlova M. (2013). The Role of the p53 Protein in the Prediction of Clinical Resistance to Cytostatic Treatment in Cancer Patients. Oncol. Rep..

[49] Yang B., Li X., Ren T., Yin Y. (2019). Autoantibodies as Diagnostic Biomarkers for Lung Cancer: A Systematic Review. Cell Death Discov..

[50] Kawada J., Saito T., Kurokawa Y., Kawabata R., Takeno A., Takeoka T., Nose Y., Wada H., Eguchi H., Doki Y. (2023). Serum NY-ESO-1 and p53 Antibodies as Useful Tumor Markers in Gastric Cancer. Ann. Gastroenterol. Surg..

[51] Colombo C., Pogliaghi G., Tosi D., Muzza M., Bulfamante G., Persani L., Fugazzola L., Cirello V. (2022). Thyroid Cancer Harboring PTEN and TP53 Mutations: A Peculiar Molecular and Clinical Case Report. Front Oncol..

[52] Gąsior-Perczak D., Kowalik A., Kopczyński J., Macek P., Niemyska K., Walczyk A., Gruszczyński K., Siołek M., Dróżdż T., Kosowski M. (2024). Relationship between the Expression of CHK2 and p53 in Tumor Tissue and the Course of Papillary Thyroid Cancer in Patients with *CHEK2* Germline Mutations. Cancers.

[53] Vrinceanu D., Dumitru M., Marinescu A., Serboiu C., Musat G., Radulescu M., Popa-Cherecheanu M., Ciornei C., Manole F. (2024). Management of Giant Thyroid Tumors in Patients with Multiple Comorbidities in a Tertiary Head and Neck Surgery Center. Biomedicines.

[54] Peuget S., Zhou X., Selivanova G. (2024). Translating p53-Based Therapies for Cancer into the Clinic. Nat. Rev. Cancer.

[55] Chai D., Wang J., Fan C., Lim J.M., Wang X., Neeli P., Yu X., Young K.H., Li Y. (2024). Remodeling of Anti-Tumor Immunity with Antibodies Targeting a p53 Mutant. J. Hematol. Oncol..

