# Supplementary material for: Rationale for Testing TP53 Mutations in Thyroid Cancer—Original Data and Meta-Analysis
Source: Int J Mol Sci. 2025 Jan 25;26(3):1035. doi: 10.3390/ijms26031035 (PMC11817394; doi:10.3390/ijms26031035)
Supplement: Supplementary file 1 [file ijms-26-01035-s001.zip › ijms-3368318-supplementary.pdf]

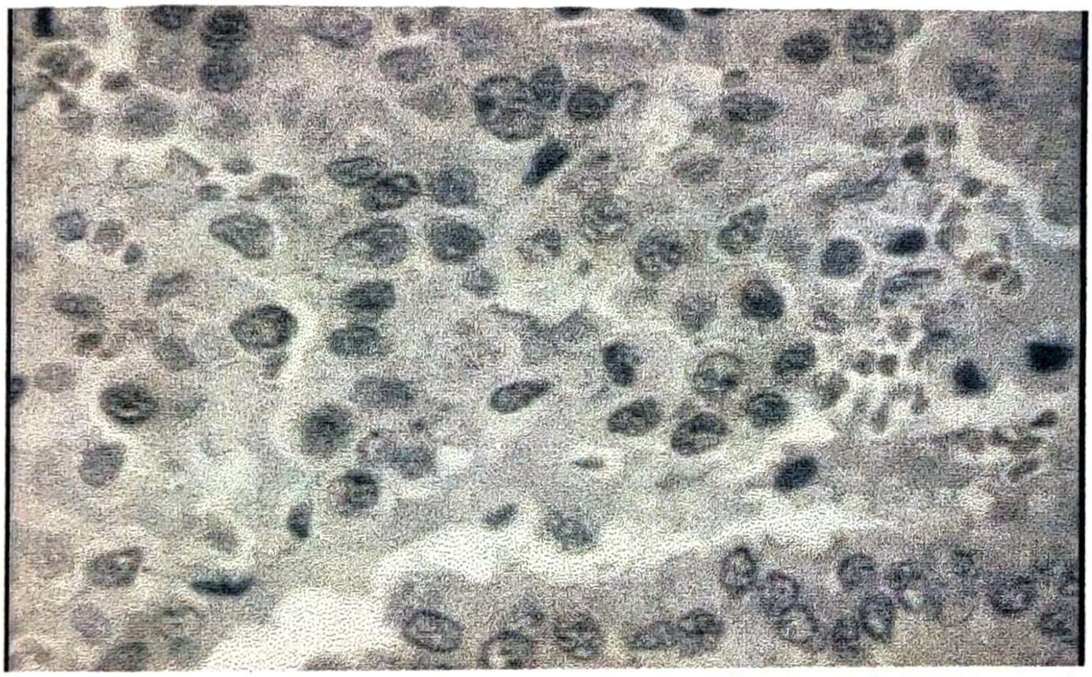

Figure S1. Papillary carcinoma - overexpression assessment - (+); magnification 40x.

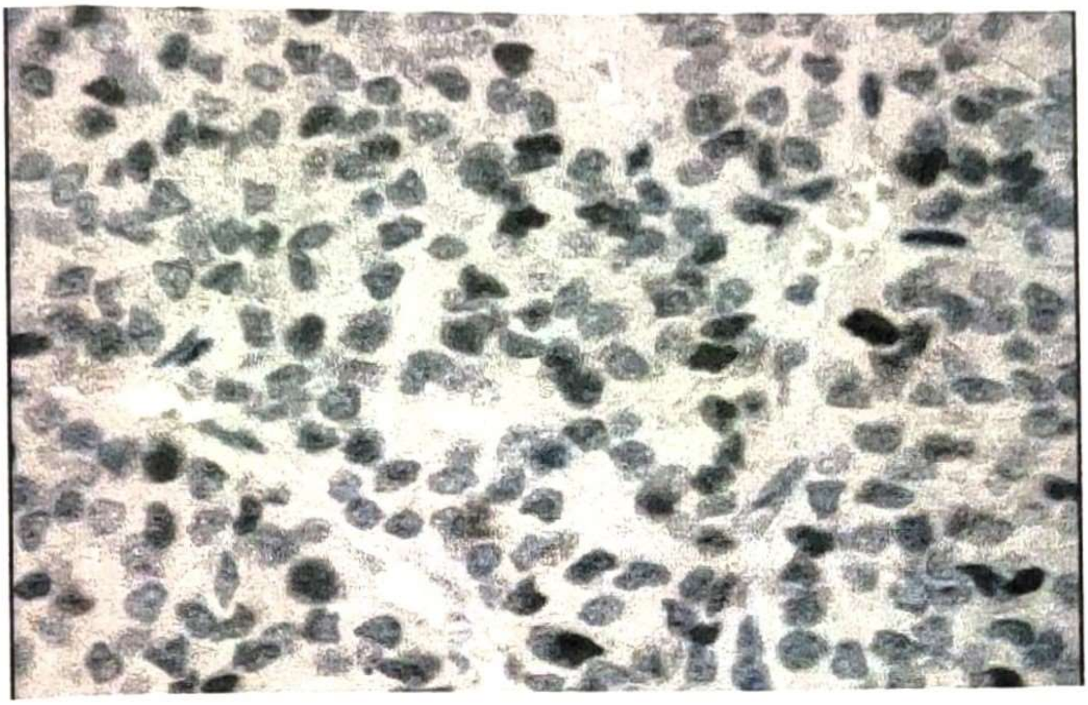

Figure S2. Folicular carcinoma - overexpression assessment - (++); magnification 40x.

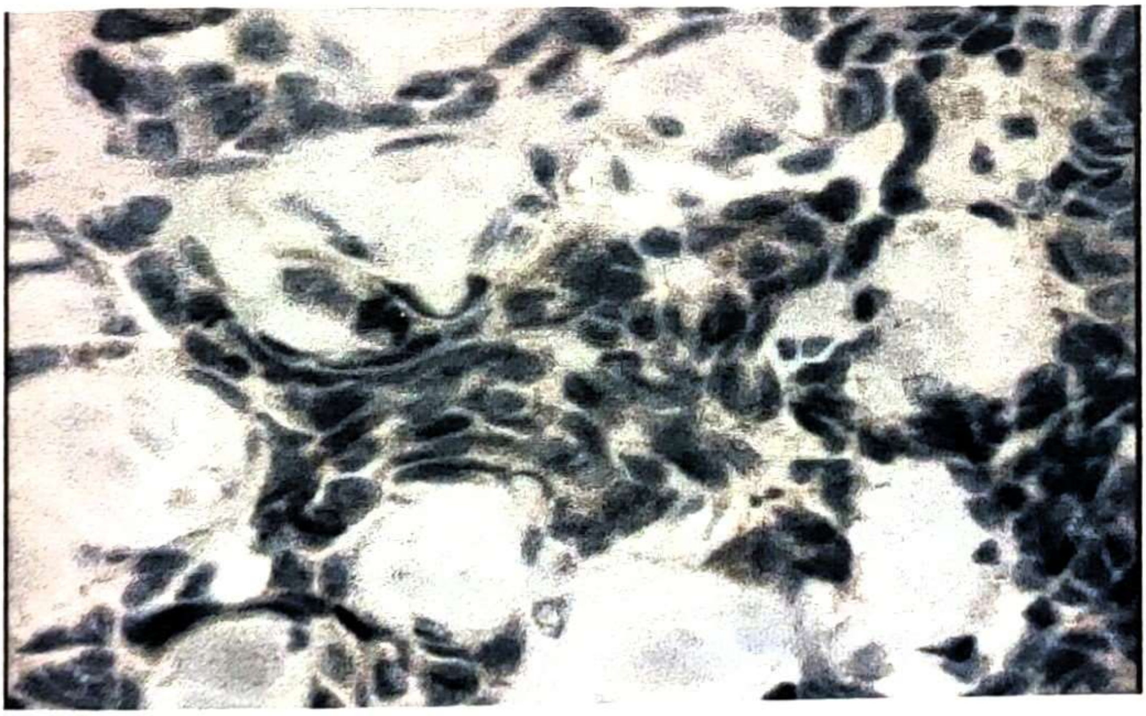

Figure S3. Anaplastic carcinoma - overexpression assessment - (+++); magnification 40x.
